# Dissipated energy during protective mechanical ventilation

**DOI:** 10.1186/2197-425X-3-S1-A663

**Published:** 2015-10-01

**Authors:** M Gotti, M Cressoni, D Chiumello, C Chiurazzi, I Algieri, M Brioni, M Amini, D Massari, A Cammaroto, MT Guanziroli, C Montaruli, K Nikolla, L Gattinoni

**Affiliations:** Università degli Studi di Milano, Dipartimento di Fisiopatologia Medico-Chirurgica e dei Trapianti, Milano, Italy

## Introduction

From literature we know that a cornerstone of the protective lung ventilation in Acute Respiratory Distress Syndrome (ARDS) patients[1] and during general anesthesia[2] is a low tidal volume. On the other hand, driving pressure seems to be the variable that best stratifies mortality risk[3]. Our hypothesis is that the combination of volume and pressure, that is the energy dissipated into respiratory system, is the main determinant of a ventilator-induced lung injury (VILI).

## Objectives

To measure dissipated energy into the respiratory system during mechanical ventilation, maintaining the same minute ventilation in each patient, setting tidal volume of 6 ml/kg or 12 ml/kg and PEEP 5 cmH_2_O.

## Methods

All patients were deeply sedated, curarized, intubated and ventilated. After a recruitment maneuver, ventilatory parameters (in a random order 6 or 12 ml/kg, PEEP 5 cmH_2_O, and the respiratory rate needed to maintain the same minute ventilation in the 2 conditions) were set and a recording of airways pressure and flow curves was made, in order to reconstruct the corresponding dynamic pressure-volume (PV) curve. We measured dissipated energy in each breaths by the hysteresis area of the PV curve of the respiratory system; we calculated the total dissipated energy into respiratory system multiplying energy dissipated during every breath by the respiratory rate.

## Results

We included 23 ARDS patients (PaO_2_/FiO_2_ 183 ± 75 and PEEP 9 ± 3 cmH_2_O at ICU admission), 7 obese patients (BMI 42 ± 10) and 11 patients with healthy lungs, both after elective surgery. In the 3 groups considered, the total dissipated energy is greater during 12 ml/kg if compared to 6 ml/kg ventilation, in particular: ARDS patients 7.60 [6.32-8.78] J/min VS 6.06 [4.57-7.12] J/min (p < 0.001), obese patients 8.71 [7.69-10.98] J/min VS 7.35 [6.68-10.52] J/min (p < 0.001), healthy lungs patients 5.04 [3.48-6.67] J/min VS 4.02 [3.18-4.85] J/min (p < 0.001) (Wilcoxon matched pairs test). The same minute ventilation was maintained increasing respiratory rate: from 9 [8-9] to 18 [17-20] breaths/min in ARDS patients (p < 0.001), from 8 [7-10] to 18 [14-20] breaths/min in healthy lungs patients (p = 0.004) and from 10 [9-10] to 20 [20-21] in obese patients (p = 0.02) (Wilcoxon matched pairs test).

## Conclusions

At the same minute ventilation, in the same patient, tidal volume of 6 ml/kg protects respiratory system from an excess of dissipated energy, despite the increase in respiratory rate; these results were observed in ARDS patients and in patients under general anesthesia, including the obese ones. The relationship between tidal volume and dissipated energy in the clinical range seems to be not linear.
